# Physiological signature of a novel potentiator of AMPA receptor signalling

**DOI:** 10.1016/j.mcn.2018.07.003

**Published:** 2018-10

**Authors:** Blanka R. Szulc, Stephen T. Hilton, Arnaud J. Ruiz

**Affiliations:** Department of Pharmacology, School of Pharmacy, University College London, London, UK

## Abstract

We have synthesized a novel small molecule based on the pyrrolidinone–containing core structure of clausenamide, which is a candidate anti–dementia drug. The synthetic route yielded multi–gram quantities of an isomeric racemate mixture in a short number of steps. When tested in hippocampal slices from young adult rats the compound enhanced AMPA receptor–mediated signalling at mossy fibre synapses, and potentiated inward currents evoked by local application of l–glutamate onto CA3 pyramidal neurons. It facilitated the induction of mossy fibre LTP, but the magnitude of potentiation was smaller than that observed in untreated slices. The racemic mixture was separated and it was shown that only the (−) enantiomer was active. Toxicity analysis indicated that cell lines tolerated the compound at concentrations well above those enhancing synaptic transmission. Our results unveil a small molecule whose physiological signature resembles that of a potent nootropic drug.

## Highlights

• A small molecule was developed using a novel approach towards the synthesis of clausenamide, which is thought to have anti–dementia properties.
• When tested in hippocampal slices the compound enhanced AMPA receptor signalling as well as glutamate–evoked currents in CA3 pyramidal neurons.
• It facilitated the induction of mossy fibre LTP.
• Cytotoxicity experiments revealed no deleterious effect to cells at sub–millimolar concentrations.

## 1 Introduction

Diseases associated with memory impairment pose a growing challenge worldwide due to an ageing population (Shah et al., 2016). The current clinical approach taken to impede cognitive decline in patients diagnosed with dementia advocates the use of drugs that enhance the state of vigilance while improving memory and decision–making (Lynch and Gall, 2013; Iwata et al., 2015; Partin, 2015). A variety of such drugs act by enhancing synaptic pathways from converging cholinergic and dopaminergic systems into the forebrain, or by raising endogenous catecholamine, orexin, and histamine levels. In addition, drugs that directly bind to glutamate receptors have been designed to enable a more selective intervention on excitatory circuits. In particular, the alpha–amino–3–hydroxy–5–methyl–4–isoxazole propionic acid (AMPA) receptor represents a major target for drug discovery because of its critical role in synaptic plasticity, the cellular mechanisms of which are thought to underlie learning and memory, including long term potentiation (LTP) and long term depression (LTD) (Huganir and Nicoll, 2013). However, despite decades-long pursuit to identify positive AMPA receptor modulators (PAMs) and bring them to clinic, the interest for such compounds is waning, as clinical trials fail to show efficacy or avoid significant toxicity. There is thus urgent need to develop new compounds that are highly potent at low concentrations, enhancing target selectivity while minimizing side effects.

Recently, we synthesized a new heterocyclic compound named BRS–015 (Szulc et al., 2015). BRS–015 is a low molecular weight 5-membered heterocycle based on the pyrrolidone core related to the structure of other members of the racetam family. The compound contains a single chiral centre and is synthesized as a racemic mixture. BRS–015 is structurally related to the excitotoxin – kainic acid, a potent AMPA/kainate receptor agonist, and to the natural product clausenamide extracted from the shrub *Clausena lansium* used in Chinese folk medicine. Clausenamide has been shown to enhance LTP (Feng et al., 2009) and is thought to possess anti–dementia properties (Chu and Zhang, 2014; Chu et al., 2016).

Here, we focused on the physiological and toxicology profile of BRS–015. We asked whether our lead compound modulated synaptic transmission and synaptic plasticity in the hippocampus. We chose to study the actions of BRS–015 at hippocampal mossy fibre synapses because they express the three main types of ionotropic glutamate receptors whose currents can be studied in isolation in CA3 pyramidal neurons. These synapses also exhibit a form of LTP whose expression is independent of *N*–methyl–d–aspartate (NMDA) receptor activation (Yamamoto et al., 1992; Zalutsky and Nicoll, 1992; Yeckel et al., 1999; Reid et al., 2004). Finally, mossy fibres convey a strong excitatory drive to CA3 pyramidal neurons that is believed to be responsible for processing and encoding distinct contextual associations arising in the dentate gyrus (Jones et al., 2016; Ruiz and Kullmann, 2012; Stella et al., 2013). We find that our small molecule acts as a powerful enhancer of AMPA receptor–mediated synaptic transmission at mossy fibre synapses. In addition, we demonstrate that a single enantiomer is active and that the racemic mixture eases mossy fibre LTP induction. Remarkably, we also find it well tolerated by cells, highlighting that it is a good potential candidate for further pre–clinical development.

## 2 Results

### 2.1 BRS–015 induced enhancement of dentate–CA3 synaptic transmission

**Fig. 1:**
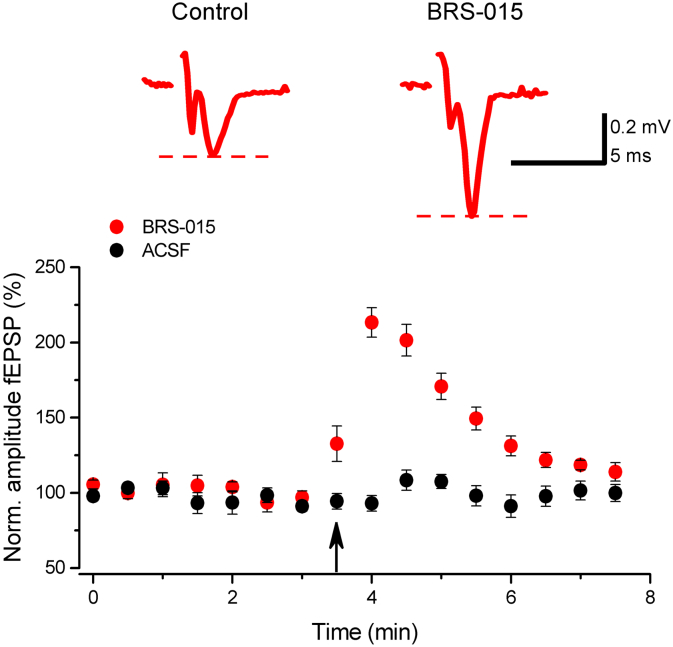
Application of BRS–015 in stratum lucidum potentiates dentate–evoked fEPSPs in CA3. In red is the fEPSP amplitude plotted against time showing a rapid and reversible increase following the pressure application of BRS–015 (113.1 ± 22.8%, n = 5, *P* = 0.007). In black, no significant increase in fEPSP amplitude after local application of ACSF. Data are from 5 slices. Sample traces show fEPSPs from one experiment (stimulus artifacts removed for clarity). Picrotoxin (100 μM), CGP–52432 (5 μM) and d–AP5 (50 μM) are present in the bathing solution. Arrow indicates the onset of BRS–015 or ACSF puffs. Circles represent the mean fEPSP amplitude. Error bars: SEM. (For interpretation of the references to colour in this figure legend, the reader is referred to the web version of this article.)

**Supplementary Scheme 1:**
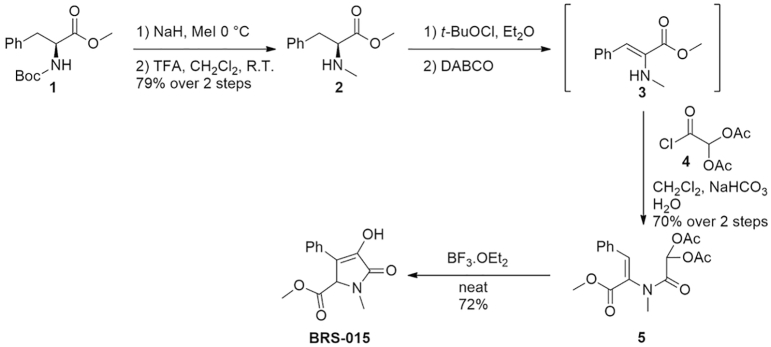
Synthesis of BRS–015. *N*–Boc–phenylalanine methyl ester was *N*–methylated and deprotected to generate *N*–methylphenylalanine methyl ester (**2**) in 79% yield over two steps. Dehydration using *tert*–butyl hypochlorite and acylation with diacetoxyacetyl chloride (**5**) followed by cyclisation in neat boron trifluoride diethyl etherate gave BRS–015 in 72% yield.

BRS–015 was obtained using a novel approach towards the synthesis of the core structure of clausenamide *via* an intramolecular acylal cyclisation (Szulc et al., 2015) (Supplementary Scheme 1). We first asked whether BRS–015 applied in the region of mossy fibre synapses had an effect on field EPSPs (fEPSPs) recorded in CA3. We positioned a bipolar tungsten electrode in stratum granulosum in the dentate gyrus to deliver electrical stimuli every 10 s. The recording pipette was placed in stratum lucidum in CA3, 100–150 μm away from a pressure–application pipette filled with BRS–015 (1 mM). With this arrangement we avoided manipulating axonal receptors in the vicinity of the stimulation electrode when delivering the compound. Procedures for characterization of mossy fibre responses were applied prior to blocking GABA_A_ and GABA_B_ receptors with picrotoxin (100 μM) and 3–([[[(3,4–dichlorophenyl)methyl]amino]propyl]diethoxymethyl)phosphinic acid (CGP–524332; 5 μM), respectively (see Materials and methods). Pressure application of BRS–015 (20 psi, 10 s) in stratum lucidum reversibly increased the amplitude of evoked fEPSPs (Fig. 1). After retraction and replacement of the puff pipette, application of control artificial cerebro spinal fluid (ACSF) at the same location had no effect on fEPSP amplitude, arguing against the presence of mechanical movements. These results demonstrate that the application of BRS–015 in CA3 has a powerful enhancing effect on glutamatergic transmission originating from the dentate gyrus.

**Fig. 2:**
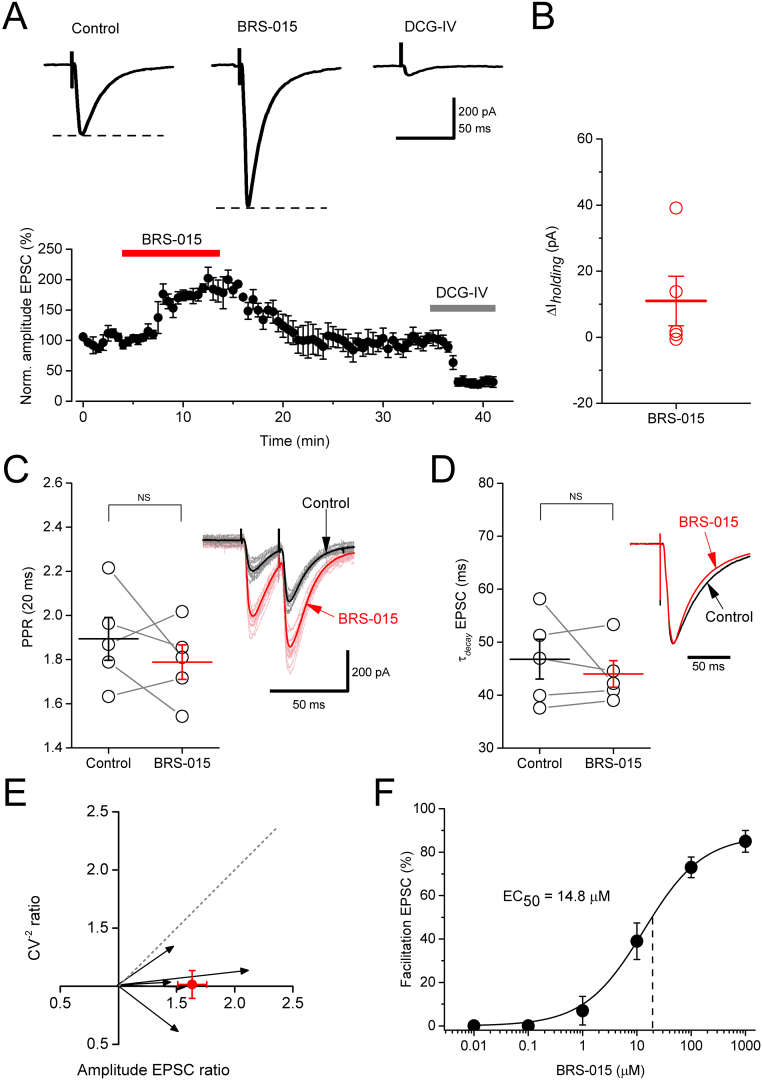
Dose–dependent effect of BRS–015 at mossy fibre synapses. A, Plot of normalized EPSC amplitude against time showing a reversible increase (63.5 ± 12.5%, *P* = 0.002) in the presence of BRS–015 (100 μM). Consecutive application of the mGluRII agonist DCG–IV (1 μM) depresses EPSCs by 70.7 ± 29.3% (*P* = 0.01). Data pooled from 5 neurons. Representative current traces for each condition are shown on top. B, No effect of BRS–015 (100 μM) on holding current (∆I_*holding*_: 10.9 ± 7.4 pA, *P* = 0.21). Horizontal bar: mean. Error bars: SEM. C, BRS–015 (100 μM) does not alter the PPR (control: 1.89 ± 0.09 *versus* BRS–015: 1.78 ± 0.07, *P* = 0.14). Sample traces show consecutive paired EPSCs in control condition (black) and in the presence of BRS–015 (red). D, The EPSC τ_*decay*_ is not affected by BRS–015 (control: 46.8 ± 3.7 ms *versus* BRS–015: 43.9 ± 2.5 ms, *P* = 0.45). Example traces show peak–scaled EPSCs from one cell, in control condition (black) and in the presence of BRS–015 (red). Each paired circle represents data from one experiment. E, in red is the mean fractional change in CV^−2^ plotted against the mean fractional change in EPSC amplitude (BRS–015/baseline ratio of CV^−2^ = 1.1 ± 0.1, n = 5; *P* = 0.9). Error bars: SEM. Vectors represent fractional changes in CV^−2^ and amplitude in individual cells. Responses on the horizontal (y = 1) line depict changes in EPSC amplitude without changes in variance and therefore represent changes in quantal size. The dashed grey line is the 45° identity line. F, Concentration–facilitation relation and fitting with a non–linear logistic function. BRS–015 (1 μM) produces a non–significant increase in EPSC amplitude (7.1 ± 6.5%, n = 4, *P* = 0.3). BRS–015 (10 μM) increases it by 35.2 ± 18.4% (n = 4, *P* = 0.15) and BRS–015 (1 mM) by 87.5 ± 5.2% (n = 4; *P* = 4.E^−4^). Error bars: SEM. Vertical dashed line indicates the EC_50_. Picrotoxin (100 μM), CGP–52432 (5 μM) and d–AP5 (50 μM) are continuously present in the bathing solution. (For interpretation of the references to colour in this figure legend, the reader is referred to the web version of this article.)

To gain insight into the mechanism involved in the BRS–015–induced enhancement, we recorded EPSCs in CA3 pyramidal neurons held in voltage–clamp with a CsCl based pipette solution (V_*holding*_ = −70 mV). Paired stimuli (50 Hz) were delivered *via* the dentate stimulus electrode every 20 s, interleaved with single stimuli every 10 s. Picrotoxin (100 μM), CGP–52432 (5 μM) and d–(−)–2–amino–5–phosphonopentanoic acid (d–AP5, 50 μM) were always present in control ACSF and BRS–015 perfusion solutions. Superfusion of slices with BRS–015 (100 μM) increased the amplitude of dentate–evoked EPSCs. The effect was fully reversible upon switching from BRS–015 to ACSF solution (Fig. 2A). The consecutive application of (2*S*,2′*R*,3′*R*)–2–(2′,3′–dicarboxycyclopropyl)glycine (DCG–IV; 1 μM) had the opposite depressant effect, consistent with the high sensitivity of mossy fibres to group II metabotropic glutamate receptor agonists. The holding current (I_*holding*_), paired–pulse ratio (PPR) of EPSC amplitude and EPSC decay–time constant (τ_*decay*_), were not altered by BRS–015 application (Fig. 2B–D). We also performed CV (coefficient of variation) analysis on evoked EPSCs. This type of analysis makes use of the inherent variability in synaptic responses over many trials, which is caused by stochastic neurotransmitter release. Changes in quantal size precisely change both the mean EPSC and the variance such that the normalized ratio of mean^2^/variance, also known as CV^−2^, remains constant. In contrast, changes in quantal content will cause proportional changes of equal magnitude in both amplitude and CV^−2^ ratios (Faber and Korn, 1991). The effect of BRS-015 on the variance of EPSCs can be visualized graphically by plotting the mean EPSC amplitude against CV^−2^ (Fig. 2E). The potentiation of evoked EPSCs by BRS–015 was accompanied by no change in the statistic CV^−2^, consistent with increased quantal size. Next, we examined the effect of BRS–015 over a range of concentrations (1 nM–1 mM) in order to characterize the concentration–facilitation relationship (Fig. 2F). Sub–micromolar concentrations of BRS–015 (0.1–1 nM) had no effect on EPSC amplitude, whereas BRS–015 (1 μM) only produced a minor increase. BRS–015 (1 mM) produced the largest increase in EPSC amplitude. Fitting of the concentration–facilitation relation with a logistic function yielded an EC_50_ of 14.8 μM. These results confirm our observations made with field electrodes and highlight the dose–dependent enhancing effect of BRS–015 on NMDA receptor–independent neurotransmission at mossy fibre synapses.

### 2.2 BRS–015 does not affect kainate receptors or NMDA receptors

**Fig. 3:**
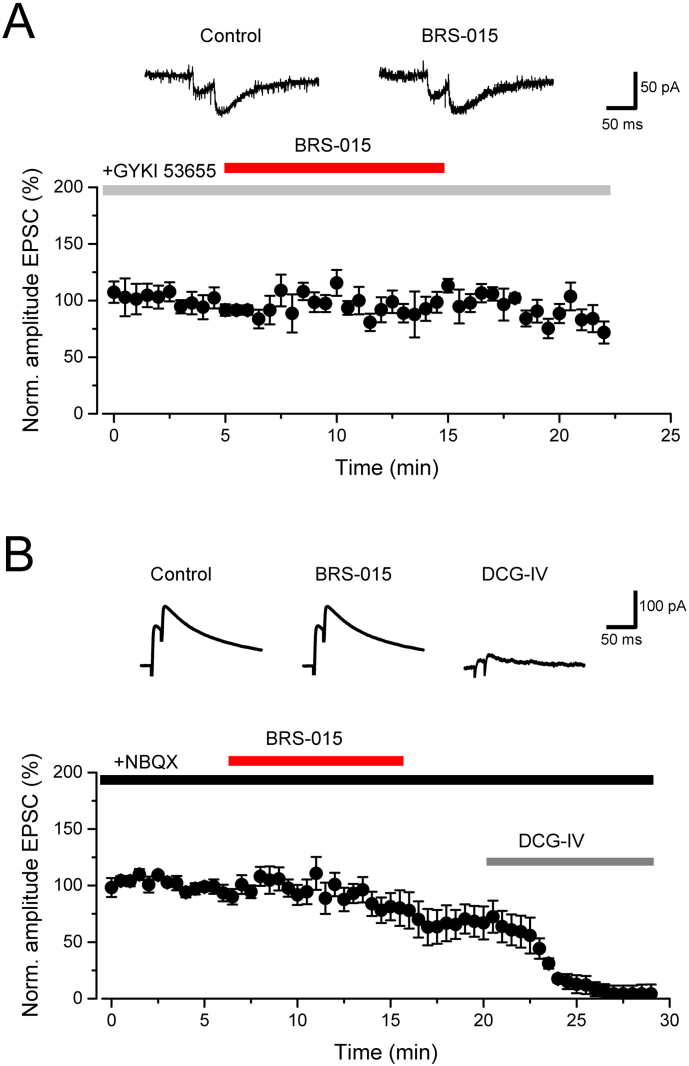
BRS–015 does not affect kainate and NMDA receptor–mediated EPSCs. A, Kainate receptor–mediated EPSCs isolated by adding picrotoxin (100 μM), CGP–52432 (5 μM), d–AP5 (50 μM), and GYKI–53655 (50 μM) to the bathing solution. Superfusion of slices with BRS–015 (100 μM) has little effect on EPSC amplitude (13.8 ± 7.7%, n = 5, *P* = 0.17). Sample current traces are shown on top (averages of 5 consecutive trials). B, Superfusion of BRS–015 (100 μM) does not affect NMDA receptor–mediated EPSCs (n = 9; *P* = 0.75), whereas application of DCG–IV (1 μM) depresses them by 92.2 ± 0.5% (n = 6; *P* = 0.01). Each point represents the mean current amplitude. Error bars: SEM. NBQX (20 μM), picrotoxin (100 μM) and CGP–52432 (5 μM) are present in the bathing solution.

Because in our experiments NMDA receptors were blocked, we asked the question whether the enhancing effect of BRS–015 solely involved AMPA receptors, kainate receptors, or both (Cossart et al., 2002; Mulle et al., 1998; Vignes and Collingridge, 1997; Castillo et al., 1997). We first examined kainate receptor–mediated EPSCs in the presence of GABA receptor blockers, d–AP5 (50 μM), and the AMPA receptor antagonist 1–(4–aminophenyl)–3–methylcarbamyl–4–methyl–3,4–dihydro–7,8–methylenedioxy–5*H*–2,3–benzodiazepine (GYKI–53655, 50 μM). Superfusion of BRS–015 (100 μM) had no effect on kainate receptor–mediated EPSCs (Fig. 3A). In a separate set of experiments, we recorded NMDA receptor–mediated EPSCs at V_*holding*_ = +40 mV, in the presence of picrotoxin (100 μM), CGP 52432 (5 μM) and 2,3–dioxo–6–nitro–1,2,3,4–tetrahydrobenzo[*f*]quinoxaline–7–sulfonamide (NBQX, 20 μM). Although in these conditions evoked EPSCs tended to run down, superfusion of BRS–015 (100 μM) had no major effect on their amplitude (Fig. 3B). Thus, neither NMDA nor kainate receptor–mediated EPSCs were sensitive to BRS–015 implying that it might selectively enhance AMPA receptors.

### 2.3 BRS–015 does not affect basic electrical membrane properties

**Fig. 4:**
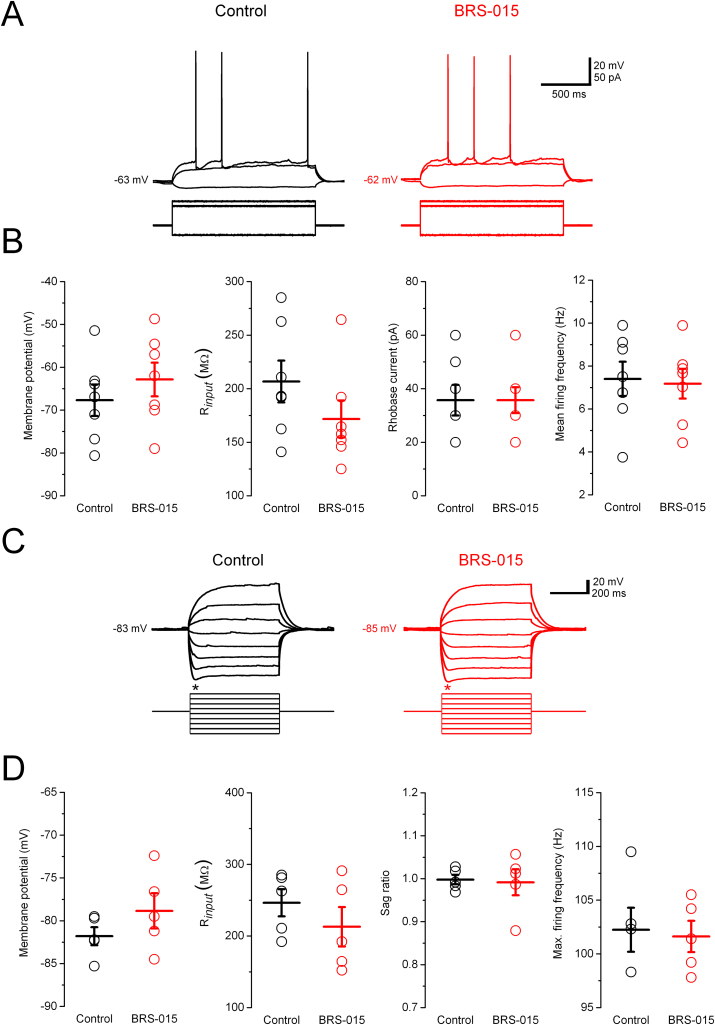
BRS–015 does not affect the electrical membrane properties of CA3 pyramidal neurons and dentate granule cells. A, Voltage deflections recorded in a CA3 pyramidal neuron in response to hyperpolarizing and depolarizing current steps (−20–50 pA, 1 s), in control condition (black) and in the presence of BRS–015 (100 μM, red). There are no significant changes in membrane potential, R_*input*_, rheobase current, and firing. B, Summary data for membrane potential (control: −67.7 ± 3.6 mV *versus* BRS–015: −62.9 ± 3.9 mV, *P* = 0.2); R_*input*_ (control: 206.7 ± 19.5 MΩ *versus* BRS–015: 171.7 ± 17.3 MΩ, *P* = 0.053); rheobase current (control: 35.7 ± 5.7 pA *versus* BRS–015: 35.7 ± 4.8 pA); mean firing frequency (control: 7.4 ± 0.8 Hz *versus* BRS–015: 7.1 ± 0.7 pA); maximum firing frequency (control: 12.2 ± 1.5 Hz *versus* BRS–015: 13.5 ± 2.1 Hz, *P* = 0.21). Data pooled from 7 neurons. C, Voltage deflections recorded in a dentate granule cell in response to hyperpolarizing and depolarizing current steps (−20–50 pA, 1 s), in control condition (black), and in the presence of BRS–015 (100 μM, red). Note the presence of a “sag” at hyperpolarized potential (asterisk). D, Summary data for membrane potential (control: −81.8 ± 1.1 mV *versus* BRS–015: −78.8 ± 2.1 mV, *P* = 0.2); R_*input*_ (control: 246.4 ± 18.9 MΩ *versus* BRS–015: 212.8 ± 27.6 MΩ, *P* = 0.15); sag ratio (control: 0.99 ± 0.01 *versus* BRS–015: 0.99 ± 0.03 pA, *P* = 0.83); maximum firing frequency (control: 102.2 ± 2.1 Hz *versus* BRS–015: 101.6 ± 1.5 Hz, *P* = 0.66). Data pooled from 5 granule cells. Each circle represents the data from one experiment. Horizontal bar: mean. Error bars: SEM. The bathing solution contains picrotoxin (100 μM), CGP–52432 (5 μM) and D–AP5 (50 μM). (For interpretation of the references to colour in this figure legend, the reader is referred to the web version of this article.)

BRS–015–induced enhancement of transmission could result from increased excitability of CA3 pyramidal neurons (*e.g.*, *via* a global change in R_*input*_). Hence, recordings were undertaken in current–clamp mode to analyse possible changes in the basic electrical membrane properties of neurons directly involved in the dentate–CA3 connection. I–V relations were obtained in CA3 pyramidal neurons and granule cells by delivering a series of hyperpolarizing and depolarizing current steps, in control condition, and in the presence of BRS–015 (Fig. 4). Superfusion of BRS–015 (100 μM) did not alter the membrane potential or the R_*input*_ of CA3 pyramidal neurons. In addition, BRS–015 had no effect on the firing frequency (Fig. 4A,B). Similar conclusions were reached in granule cells, where application of BRS–015 (100 μM) neither modified the R_*input*_ nor the membrane potential, the sag ratio or the maximum firing rate (Fig. 4C,D). Thus, BRS–015 does not promote the depolarizing and shunting effect of a typical ionotropic glutamate receptor agonist, implying that it does not directly activate a membrane conductance.

### 2.4 BRS–015 potentiates glutamate–evoked currents in CA3 pyramidal neurons

**Fig. 5:**
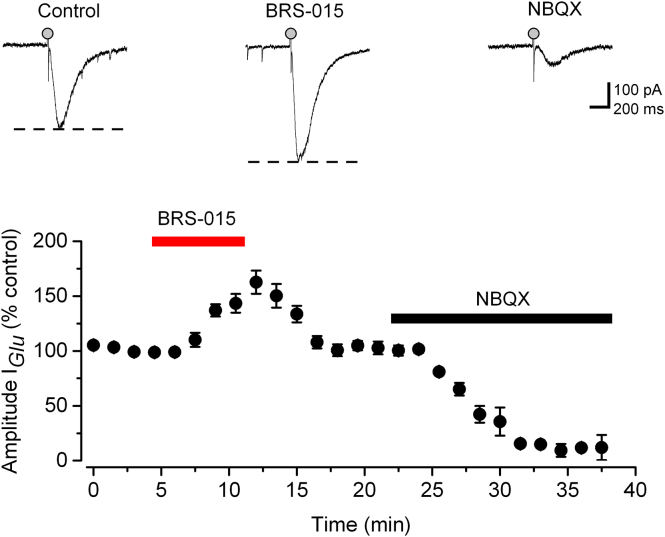
BRS–015 potentiates glutamate–evoked currents in CA3 pyramidal neurons. Amplitude of puff–evoked glutamatergic currents plotted against time showing a reversible increase (57.6 ± 15.2%, n = 4, *P* = 0.03) in the presence of BRS–015 (100 μM). Application of NBQX (20 μM) at the end of the experiment strongly depresses the currents, leaving a small residual component. Traces on top show glutamatergic currents (single trials) from one neuron, in control condition and in the presence of BRS–015 (100 μM). Glutamate puff: 5–20 psi, 10–50 ms, every 60 s (grey circle). Error bars: SEM.

The results presented so far suggest that BRS–015 does not have major presynaptic actions. Assuming that BRS–015 acts on postsynaptic targets, it should enhance glutamate–evoked currents in CA3 pyramidal neurons. We pressure–applied l–glutamate (100 μM) in the region of the apical dendrite of a patched CA3 pyramidal neuron, and recorded the resulting inward currents. GABA and NMDA receptors were blocked with picrotoxin, CGP–52432 and d–AP5, respectively. As shown in Fig. 5, superfusion of slices with BRS–015 (100 μM) reversibly increased the amplitude of glutamatergic currents. The time course of the BRS-015 enhancement of puff-evoked glutamatergic currents developed slowly and the effect was smaller compared with the potentiation of synaptic responses. Application of NBQX (20 μM) at the end of the experiments largely reduced these currents indicating that they were mostly mediated by AMPA receptors. These findings demonstrate that the BRS–015 mediated enhancement of AMPA receptor function is initiated in CA3 pyramidal neurons.

### 2.5 A single enantiomer of BRS–015 is active: comparison with piracetam

**Fig. 6:**
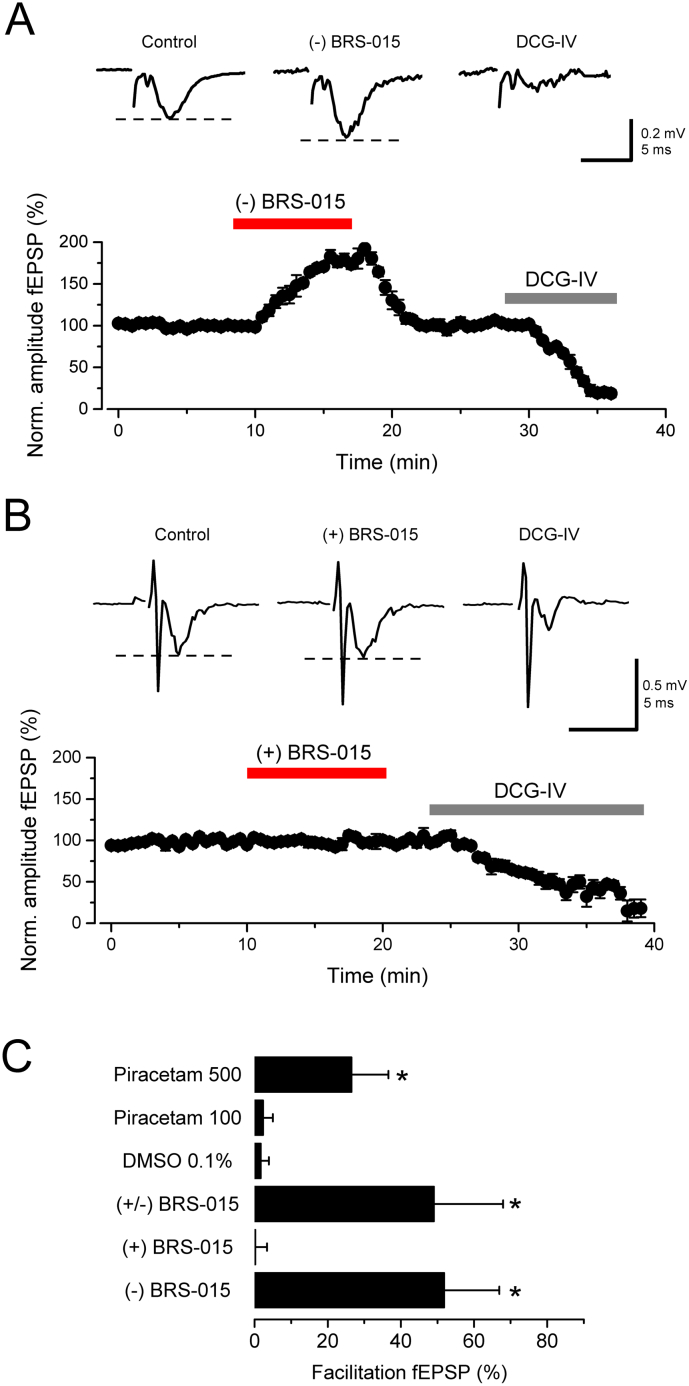
(−) BRS–015 is more potent than piracetam at low concentration. A, Plot of EPSP amplitude against time showing an increase (51.8 ± 15.1%, n = 6, *P* = 0.02) in the presence of (−) BRS–015 (100 μM). The consecutive application of DCG–IV (1 μM) depresses fEPSPs by 91.1 ± 7.2% (n = 5; *P* = 2E^−4^). Example voltage traces from one experiment are shown on top for each condition (stimulation artifacts truncated for clarity). B, Superfusion of (+) BRS–015 (100 μM) has no significant effect on fEPSP amplitude. DCG–IV (1 μM) depresses fEPSPs by 83.9 ± 11.3% (n = 5; *P* = 0.04). Sample traces show fEPSPs from one experiment (stimulus artifact removed for clarity). C, Summary data for (+/−) BRS–015 (100 μM): 49 ± 18.9%, n = 5, *P* = 0.04; DMSO 0.1%: 1.7 ± 2.2%, n = 2; piracetam (100 μM): 2.3 ± 6.7%, n = 3; piracetam (500 μM): 26.5 ± 10.1%, n = 3, *P* = 0.11; (+) BRS–015 (100 μM): 0.2 ± 3.2%, n = 5, and (−) BRS–015 (100 μM): 51.8 ± 15.1%, n = 6, *P* = 0.02. Error bars: SEM. *, *P* < 0.05, paired *t*–test.

BRS–015 is obtained as a racemate following intramolecular acylal cyclisation, yielding both the (+) and (−) enantiomers (Szulc et al., 2015). The enantiomers were separated by chiral high performance liquid chromatography (HPLC) and we investigated their effects on dentate–evoked fEPSPs. Superfusion of slices with (−) BRS–015 (100 μM) caused a reversible increase in fEPSP amplitude (Fig. 6A). In contrast, (+) BRS–015 (100 μM) had no effect on fEPSPs (Fig. 6B). Application of the racemic mixture (+/−) BRS–015 (100 μM) produced a robust enhancement, the magnitude of which did not differ from that attained with (−) BRS–015 (Fig. 6C). We also tested the nootropic drug piracetam. Piracetam (100 μM) was essentially inactive, with only very high concentrations (500 μM) producing a modest increase in fEPSP amplitude (Fig. 6C). These results indicate that the active enantiomer (−) BRS–015 is a powerful enhancer of synaptic transmission compared to the clinically relevant drug piracetam.

### 2.6 BRS–015 facilitates mossy fibre LTP induction

**Fig. 7:**
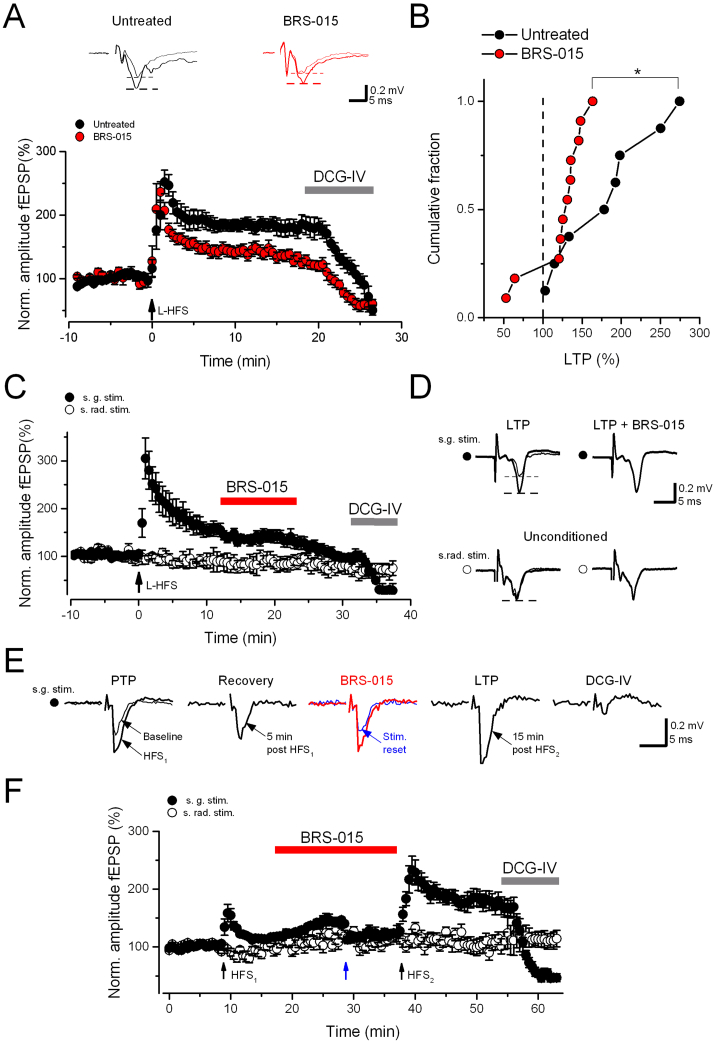
BRS–015 induced modulation of mossy fibre LTP. A, Time course of normalized fEPSP amplitude in slices continuously superfused with BRS–015 (100 μM, red) and in untreated slices (black), and depression by the mGluR II agonist DCG–IV (1 μM). There is a reduced mossy fibre LTP in the presence of BRS–015 (100 μM). B, Cumulative probability distribution of mossy fibre LTP measured as the percentage of fEPSP potentiation 15–20 min after tetanus compared with control period. LTP in slices treated with BRS–015 (111.51 ± 16.81%, n = 6) is smaller than that elicited in untreated slices (182.9 ± 20.4%, n = 8, *P* = 0.02). *, unpaired *t*–test. C, Time course of normalized fEPSP amplitude showing a non–significant increase (17.9 ± 13.5%, n = 4, *P* = 0.2) when BRS–015 (100 μM) is applied during mossy fibre LTP. fEPSPs elicited by stratum radiatum stimulation remain largely unaffected (13.3 ± 14.9% reduction, n = 4, *P* = 0.19). Addition of DCG–IV (1 μM) depresses fEPSPs evoked by stratum granulosum (s. g.) stimulation (70.9 ± 7.4%, n = 4, *P* = 0.03) but has no effect on stratum radiatum (s. rad) evoked responses. Arrow indicates the time of tetanic stimulation. D, Example traces from a single experiment depicted in panel C. E, Normalized fEPSP amplitude plotted against time showing PTP of fEPSPs (57.9 ± 12.7%, n = 11) after a stimulus burst is delivered in stratum granulosum (HFS_1_, 100 stimuli in 1 s). Subsequent superfusion with BRS–015 (100 μm) increases the amplitude of fEPSPs by 48.4 ± 9.6% (n = 11, *P* = 0.05). It has no effect on stratum radiatum evoked responses (6.2 ± 7.6%, n = 5, *P* = 0.3). Blue arrow indicates the time of a stimulus intensity reset at the dentate electrode when the enhancing effect of BRS–015 reaches a plateau. A second stimulus burst (HFS_2_, identical to HFS1) delivered after 20 min of application of BRS–015 leads to early LTP (174.3 ± 15.1%, n = 6, *P* = 0.04). Final application of DCG–IV (1 μM) depresses stratum granulosum evoked fEPSPs by 82.8 ± 3.8% (n = 4, *P* = 0.008) and has no effect on those elicited by stratum radiatum stimulation. Each point represents the mean. Error bars: SEM. Picrotoxin (100 μM), CGP–52432 (5 μM) and d–AP5 (50 μM) are included in the perfusion solution. (For interpretation of the references to colour in this figure legend, the reader is referred to the web version of this article.)

Does the effect of BRS–015 relate to LTP? We began addressing this question by inducing LTP in slices incubated with the compound and continuously superfused with it. Mossy fibre inputs were monitored with field electrodes. Picrotoxin (100 μM), CGP–52432 (5 μM) and d–AP5 (50 μM) were added to the perfusion solution to block GABA_A_, GABA_B_, and NMDA receptors. NMDA receptor–independent LTP was induced by long high–frequency stimulation delivered in stratum granulosum (l–HFS). The magnitude of LTP in slices treated with BRS–015 was significantly smaller than that elicited in untreated slices (Fig. 7A,B). We also performed experiments where BRS–015 was applied when mossy fibre synapses were already undergoing LTP. In these experiments, a second stimulus electrode was placed in the distal region of stratum radiatum to activate associational/commissural (A/C) synapses, acting as a control pathway. Application of BRS–015 (100 μM) 10 min after induction of mossy fibre LTP had no potentiating effect on dentate–evoked fEPSPs (Fig. 7C,D). Interestingly, BRS–015 did not affect synaptic transmission at A/C synapses (Fig. 7C). Finally, instead of using tetanic stimulation that induced saturating levels of LTP, we applied a milder tetanus and asked if application of BRS–015 could help it in triggering LTP. As shown in Fig. 7F application of a high–frequency stimulus burst in stratum granulosum (HFS_1_, 100 pulses for 1 s) caused a transient increase in fEPSP amplitude that decayed to near baseline level after 5 to 7 min, akin to post–tetanic potentiation (PTP). Consecutive to PTP, the superfusion of slices with BRS–015 (100 μM) increased the amplitude of dentate–evoked fEPSPs. Again, it had no effect on A/C responses indicating a degree of pathway selectivity. When a second high–frequency stimulus burst (HFS_2_) was delivered in stratum granulosum, the amplitude of fEPSPs measured 15 min after HFS_2_ was persistently increased, consistent with LTP. This larger and sustained form of potentiation contrasted with the PTP elicited by HFS_1_. Altogether, these results demonstrate that priming the tissue with BRS–015 facilitates LTP induction at mossy fibre synapses. However, the magnitude at which LTP is expressed in the presence of BRS–015 is smaller when compared to LTP in untreated tissue.

### 2.7 Toxicity profile of BRS–015 in stable cell lines

Toxicity studies were carried out in murine 3 T6 cells and human HepG2 cells. BRS–015 was shown to be essentially non-toxic in the 3 T6 cell line (2 mM maximum concentration, n = 6) and exhibited an LD_50_ of 914 μM in the HepG2 cell line (n = 6). This concentration was >50–fold higher than the observed EC_50_ for enhancement of glutamatergic transmission. Thus, BRS–015 appears non–toxic to cells at sub–millimolar concentration highlighting a good potential for physiological and therapeutic applications.

## 3 Discussion

We have examined the effects of a newly synthesized small molecule on glutamatergic transmission and found a dose–dependent enhancement of AMPA receptor–mediated signalling at mossy fibre synapses. We also showed that a single enantiomer was active and that the racemic mixture facilitated the induction of mossy fibre LTP. Finally, we found it non–toxic to cells emphasizing a good potential for further chemistry and pre–clinical development.

### 3.1 BRS–015 enhancement of basal synaptic transmission

We report the effect of BRS–015 in CA3 pyramidal neurons and found that one consequence of its activity was increased AMPA EPSC amplitude. This might have resulted from a number of steps directly or indirectly enhancing AMPA receptor function. Indeed, the compound spared kainate and NMDA receptors. However, we did not show a direct modulation of AMPA receptors, as this would have required the pulling of excised patches and fast application techniques. Nor can we rule out that metabotropic glutamate receptors may have contributed to the BRS–015 induced enhancement, as these were left unblocked. We have nevertheless several reasons to believe that BRS–015 modulated postsynaptic sites, and ultimately AMPA receptors. Firstly, blocking AMPA receptors with GYKI–53655 abolished the BRS–015 enhancement of NMDA receptor–independent synaptic transmission. Second, the compound had no effect on the PPR and statistical CV^−2^ suggesting no major change in glutamate release probability and quantal content during the potentiation of transmission. (However, these measures might not change detectably with modest increases in release probability given the high degree of variability observed with mossy fibre EPSCs.) Thirdly, BRS–015 potentiated non–NMDA currents evoked by focal application of l–glutamate onto CA3 pyramidal cells, consistent with a postsynaptic locus of action. Thus, BRS–015 did not show the physiological signature of phorbol esters whose effects are known to enhance glutamate release (Honda et al., 2000; Kamiya et al., 1988; Weisskopf et al., 1994). Nor did it mimic the previously described biphasic effect of kainic acid on mossy fibre transmission to CA3 pyramidal neurons (Schmitz et al., 2000, Schmitz et al., 2001).

The BRS-015 enhancement of puff-evoked glutamatergic currents was on average ~40% smaller than the potentiation of synaptic responses (fEPSPs or EPSCs). Such discrepancy could result from the fact that pressure-applied L-glutamate activated a mixed population of receptors, some of which were insensitive to BRS-015. In support of this, we found that BRS-015 was relatively inefficient at A/C synapses. Furthermore, the time course of enhancement during bath application of BRS-015 was slow (5–10 min) compared to rapid action (1–2 min) resulting from pressure-application of the compound (Fig. 5 *versus* Fig. 1B). The rapid time course is consistent with quick delivery of the compound in the region of synapses, as opposed to bath application, which is expected to slowly build the concentration within the slice.

### 3.2 Putative mechanisms of action of BRS-015

So how could BRS–015 possibly work? It might be that BRS–015 binds to the AMPA receptor and stabilizes the channel in an open configuration, hence retarding the onset of receptor desensitization or deactivation. Such a mechanism has been shown to underlie the actions of aniracetam (Lawrence et al., 2003; Xiao et al., 1991; Isaacson and Nicoll, 1991; Ito et al., 1990; Partin et al., 1996) and other AMPAkines (Lauterborn et al., 2016; Arai et al., 2000). However, in stark contrast with the latter benzamides, BRS–015 did not prolong τ_*decay*_ of evoked EPSCs, suggesting no major action on AMPA receptor desensitization. Alternatively, BRS–015 could be enhancing the conductance of AMPA receptors by means of phosphorylation leading to potentiation of synaptic currents (Lisman et al., 2002). Indeed, neuronal AMPA receptors contain stargazin–like auxiliary subunits known as transmembrane AMPA receptor regulatory proteins (TARPs). TARPs mediate AMPA receptor surface expression and synaptic clustering (Tomita et al., 2006) and also modulate AMPA receptor channel gating by slowing desensitization and deactivation (Tomita et al., 2005; Priel et al., 2005). The recent evidence that Ca^2+^/calmodulin–dependent protein kinase II (CaMKII) directly phosphorylates TARPγ–8 (Park et al., 2016) would lend support to this notion of BRS–015 stimulation of kinase activity. The compound would have to initiate a rise in postsynaptic Ca^2+^, either *via* l–type Ca^2+^ channels (Yeckel et al., 1999; Wang et al., 2004; Kapur et al., 1998), or following the release of Ca^2+^ from intracellular stores and mitochondria. Interestingly, signalling pathways invoking the activation of CaMKII or phospholipase C (PLC) and leading to phosphorylation of extracellular signal-regulated kinase (ERK) and cAMP responding element–binding (CREB), are thought to underlie the nootropic effects of (−)-clausenamide (Chu et al., 2016; Tang and Zhang, 2004). Finally, BRS–015 could work *via* direct stimulation of kinase activity or inhibition of phosphodiesterase activity leading to increased AMPA receptor function. In particular, weak activation of protein kinase A, which shows a similar degree of synaptic specificity and effect on EPSC amplitude, could underlie some of the effect of BRS-015.

The lack of effect of BRS–015 on stratum radiatum evoked responses remains puzzling. It could reflect a distinct AMPA receptor subunit composition between mossy fibre and A/C synapses, or different levels of basal phosphorylation of AMPA receptors. Asymmetrical synapses between large mossy fibre terminals and thorny excrescences in CA3 pyramidal neurons contain an average number of AMPA receptors exceeding 4 times the number reported for C/A synapses (Fabian-Fine et al., 2000; Nusser et al., 1998). There is also a difference in the variability of receptor content, with mossy fibre synapses having the smaller variability, whereas C/A synapses can be void of AMPA receptors (Nusser et al., 1998; Kullmann, 1994; Liao et al., 1995). Furthermore, although the AMPA receptor subunits GluA1–GluA4 are enriched both at A/C and mossy fibre synapses, higher levels of expression of GluA1 have been reported for A/C synapses (Baude et al., 1995). The number and variability in AMPA receptor expression is thus remarkably different at these two functionally distinct synapse populations, which could explain some of the specificity in the effect of BRS–015.

### 3.3 Comparison between BRS-015 and clausenamide

Naturally occurring clausenamide is in racemic form, and like BRS-015, it acts as a LTP enhancer (Chu and Zhang, 2014; Chu et al., 2016; Xu et al., 2005). Both compounds are roughly equiactive with EC50 values in the range of 10–20 μM. There are four chiral centres in clausenamide implying that sixteen stereoisomers can occur (Xu et al., 2005). The single asymmetry centre of BRS-015 seems to be a clear advantage in this regard, offering a much simpler strategy in search for analogues. In addition, (−)-clausenamide crosses the blood brain barrier and conveys most biological activity, whereas (+)-clausenamide is inactive (Ning et al., 2012). Our compound shows strikingly similar stereo-selectivity but we do not know whether it penetrates the brain if administered *in vivo*. Finally, (−)-clausenamide is far more active than the nootropic drug piracetam (Chu et al., 2016), and so is BRS-015 (Fig. 6).

A major difference with clausenamide, however, is that 100 μM BRS-015 racemate mixture was found equiactive with the (−) BRS-015 isomer. In the case of clausenamide, the (−) isomer is 5–10 time more active than its racemic form (Chu et al., 2016). This discrepancy suggests that 100 μM (−)-BRS-015 caused a near-to-saturating potentiation of synaptic transmission. Only a full dose-response relationship for the (−)-BRS-015 isomer will enable a meaningful comparison with the racemic form of this compound.

### 3.4 BRS–015 induces a short–to–long term plasticity switch

We have further shown that mossy fibre LTP in the presence of BRS–015 was smaller than in untreated tissue, and that the compound was ineffective on synaptic responses exhibiting LTP. These observations suggest that mossy fibre LTP and the putative signalling pathways engaged by BRS–015 compete for a common pool of signalling molecules or proteins. However, in contrast to our compound, several groups have reported enhanced LTP in the CA1 region following AMPAkine treatment (Xiao et al., 1991; Arai et al., 2000; Arai and Lynch, 1998; Staubli et al., 1992). This difference with our study could be the result of different stimulation protocols used for inducing LTP (l–HFS *versus* theta–burst) in different hippocampal areas (CA1 *versus* CA3). It could also be related to the age of animals, and different levels of LTP expressed at different developmental stages. Finally, we demonstrated that tetanic stimulation eliciting PTP at mossy fibre synapses (Henze et al., 2002; Vyleta et al., 2016) was capable of triggering LTP once the compound had potentiated basal glutamatergic transmission. How this occurred remains unclear, but the postsynaptic enhancement of AMPA receptors by BRS–015 could be paramount to this plasticity switch. Noteworthy, several studies have shed light on the importance of postsynaptic Ca^2+^, mGluRs, and ephrin signalling in this phenomenon (Yeckel et al., 1999; Wang et al., 2004; Kapur et al., 1998; Armstrong et al., 2006; Contractor et al., 2002). It is thus conceivable – albeit speculative – that our compound promotes a form of chemical plasticity *via* postsynaptic modifications enhancing AMPA receptor function, or leading to the insertion of new AMPA receptors at postsynaptic densities (Sinnen et al., 2017).

Together, these data unravel a novel small molecule with a strong and reversible enhancing effect on AMPA receptor–mediated synaptic transmission. The effect was specific to mossy fibre synapses where the compound facilitated the induction of LTP. Our lead molecule thus demonstrates surprising and unexpected activity as enhancer of dentate gyrus inputs into the hippocampus, which may be useful in the treatment of memory related disorders.

## 4 Materials and methods

### 4.1 Electrophysiology

All experiments were performed on male Sprague Dawley rats (Harlan Laboratories Ltd., Oxon, UK), aged postnatal day 21–40. This study was performed in accordance with the Animals (Scientific Procedures) Act 1986. Animals were placed inside a chamber saturated with an isoflurane/O_2_ mixture and anesthetized. The level of anaesthesia was tested by a paw pinch. After decapitation, the brain was removed and kept in ice–cold dissecting solution. Transverse hippocampal slices (300 μm) were obtained using a vibratome (Leica, VT–1200S). Slices were stored at 35 °C for 30 min after slicing and then at 22 °C. For the dissection and storage of slices, the solution contained: NaCl (87 mM), NaHCO_3_ (25 mM), glucose (10 mM), sucrose (75 mM), KCl (2.5 mM), NaH_2_PO_4_ (1.25 mM), CaCl_2_ (0.5 mM) and MgCl_2_ (7 mM). During experiments, slices were superfused with artificial cerebro–spinal fluid solution (ACSF) containing: NaCl (125 mM), NaHCO_3_ (25 mM), glucose (25 mM), KCl (2.5 mM), NaH_2_PO_4_ (1.25 mM), CaCl_2_ (4 mM) and MgCl_2_ (4 mM), equilibrated with 95% O_2_/5% CO_2_. The divalent cation concentrations were kept high to suppress polysynaptic transmission. The osmolarity and pH of perfusion solutions were adjusted to ~320 mOsmol/L and 7.3, respectively. All recordings were performed at 21 °C. A slice was transferred into the recording chamber and visualized with an Olympus BX 51WI microscope (Olympus Europa Holding GmbH, Hamburg, Germany) connected to a KPM–3 Hitachi infrared video camera. A bipolar stimulating electrode (FHC Inc., Bowdoin, Maine, USA) was positioned under low magnification (10×) in the supra–granular blade of the dentate gyrus to activate mossy fibre synapses every 10–20 s using constant current (0.3–1.5 μA, 50–200 μs square pulses). To set the intensity of stimulation an input-output (I/O) relation was obtained for each slice when applying the control perfusion solution. The stimulus intensity was set such that the amplitude of the test fEPSP reached 30–40% of maximum amplitude based on the I/O curve. Similar stimulation intensities were used for regular testing of fEPSPs (Fig. 1, Fig. 6) and for HFS (Fig. 7). Two–pathway experiments were performed by positioning a second stimulating electrode in the distal part of stratum radiatum in CA3 to activate A/C synapses. A pipette filled with ACSF was inserted in stratum lucidum of the CA3 sub–region to record fEPSPs in response to electrical stimulation in the dentate gyrus. For two–pathway experiments, interleaved fEPSPs were recorded at one or the other pathway. Synaptic responses were recorded with an Axopatch 200B amplifier (Molecular Devices), filtered at 2 kHz (internal 4–pole low–pass Bessel filter), and sampled at 10 kHz. Two tests were routinely applied to verify that the signal recorded in stratum lucidum was a mossy fibre fEPSP. First, increasing the stimulation frequency caused pronounced facilitation (>2.5–fold at 1 Hz at room temperature). Second, application of the group II mGluR agonist DCG–IV (1 μM) depressed the fEPSP amplitude to <20% of control fEPSP (Salin et al., 1996).

For patch–clamp experiments, neurons were visualized under infrared–differential interference contrast (DIC) imaging with a water–immersion high–magnification (60×) objective (Olympus) and a four–fold magnification changer (Luigs & Neumann GmbH, Ratingen, Germany). The pipette solution used for voltage–clamp recordings contained: CsCl (120 mM), QX314–Br (5 mM), NaCl (8 mM), MgCl_2_ (0.2 mM), HEPES (10 mM), EGTA (2 mM), MgATP (2 mM), Na_3_GTP (0.3 mM), at pH 7.2 and osmolarity 310 mOsm/L. The pipette solution used for current–clamp recordings contained: 135 mM K–gluconate, 5 mM KCl, 1 mM CaCl_2_, 5 mM EGTA–Na, 10 mM HEPES, 10 mM glucose, 5 mM MgATP, and 0.4 mM Na_3_GTP. The access resistance, monitored throughout the experiments, was <20 MΩ and results were discarded if it changed by >20%. Junction potentials were not corrected. For glutamate puffs, L–glutamate (100 μM in ACSF) was applied at 5–20 psi (10–50 ms). Puffs were applied every 60 s to allow for glutamate clearance. The puff electrode was positioned at the border between strata lucidum and radiatum, 100–150 μm away from the soma of the recorded CA3 pyramidal cell, and along its apical dendrite. Mossy fibre LTP was induced by long high–frequency tetanic stimulation (l–HFS) represented by 100 pulses in 1 s, 3 times, separated by 10 s. Slices in which LTP experiments were performed in the presence of BRS–015 were maintained within an interface chamber on a Petri dish containing BRS–015 solution (100 μM) for at least 2 h prior to recording, and were continuously superfused with it before and after LTP induction (Fig. 7A,B).

### 4.2 Data analysis and statistics

For each recording, fEPSP or EPSC amplitude was normalized to the amplitude measured 1–3 min pre–drug application. The overall change in amplitude for ‘n’ recorded neurons was determined by averaging normalized amplitudes, then expressed as a percentage. EPSC decay–time constant was obtained by fitting the average of 5–10 consecutive trials in control condition and in the presence of BRS–015 with a single exponential function using Matlab R2010a (TheMathWorks, version 7.10). For PPR measurements of EPSC amplitude at −60 mV, interleaved responses evoked by a single stimulus were subtracted from those elicited by paired stimuli (20 ms apart). PPR was determined by dividing the peak amplitude of EPSC_2_ by that of EPSC_1_. 1/CV^2^ was calculated as (mean EPSC)^2^ / (Var_EPSC_ − Var_noise_). For each neuron recorded in current–clamp mode, the I–V relation was determined by measuring the amplitude of steady state voltage deflections elicited by a series of hyperpolarizing and depolarizing current steps (−120 pA to +100 pA; 500 ms). The mean firing frequency was calculated by dividing the number of action potentials by the duration of a supra–threshold current step that did not inactivate Na^+^ channels (400–700 pA, 500 ms). The maximum firing rate was determined by the time interval between the first and second action potentials. R_*input*_ was obtained by fitting the linear portion of the I–V relation at hyperpolarized potentials. ‘Sag’ ratios were determined as the ratio between the steady state voltage and peak voltage in response to a current injection that resulted in a membrane potential negative to −120 mV. Unless otherwise noted, we routinely applied a two–tailed paired *t*–test to test the difference between the sampled means (Gaussian data scatter). The confidence intervals were calculated using OriginPro (Originlab). Data were considered significant if *P* < 0.05. Sample traces from example recordings were obtained by averaging 5–10 consecutive trials in control condition, and 5–10 min after the debut of the application of a given drug (except for Figs. 4A,C and 5). Values are given as mean ± SEM. Error bars represent SEM.

### 4.3 Toxicity assays

10,000 CRFK cells were eluted with Roswell Park Memorial Institute (RPMI) 1640 medium supplemented with 10% fetal bovine serum 100 M/mL Glutamine and 1% v/v antibiotic/antimycotic (Gibco) onto 96–well plates and incubated for 24 h at 37 °C (5% CO_2_). The medium was then removed by vacuum and replaced with the specific dilution (1 nM–1 mM) to test the toxicity (3 × 200 μL). BRS–015 (1 nM–1 mM) was then suspended in 2% di–methylsulfoxide (DMSO) and RPMI 1640 medium supplemented with 10% fetal bovine serum 100 M/mL Glutamine and 1% v/v antibiotic/antimycotic, and diluted to the desired concentrations. After 24 h, the wells were observed and the medium removed. The cells were then suspended in RPMI 1640 phenol red–free medium (180 μL) and 3–(4,5–dimethylthiazol–2–yl)–2,5–diphenyltetrazolium bromide (MTT) (3 mg/mL), and incubated 4 h at 37 °C (5% CO_2_). The medium was then removed by vacuum and the cells were lysed with methanol (200 μL) to reveal a bright purple formazan product. The methanol–formazan absorbance was then determined at 570 nm using a BioTek Synergy HT plate reader with KC4 software. Data were expressed as the percentage of viability (normalized to cells with no exposure) of compound–treated wells compared to that of untreated control wells.

### 4.4 Drugs and chemicals

Picrotoxin was purchased from Abcam Biochemicals (Cambridge, UK) and prepared in DMSO for use at a final concentration of 100 μM. BRS–015 was prepared in DMSO at a stock concentration of 50 mM. DCG–IV, CGP–52432, and NBQX were purchased from Tocris Bioscience (Bristol, UK). l–glutamic acid, d–AP5 and GYKI 53655 were purchased from Abcam Biochemicals (Cambridge, UK). All other chemicals used for the synthesis of BRS–015 can be sourced in Szulc et al. (2015).

The following is the supplementary data related to this article.
